# In Vitro Digestibility of Starch Gel in Cooked Rice Prepared with Thermo-Reversible Short-Chain Glucan Aggregates (SCGAs)

**DOI:** 10.3390/gels11090701

**Published:** 2025-09-02

**Authors:** So-Eun Yoon, Sang-Jin Ye, Min-Seok Kim, Seon-Min Oh, Jae-Sung Shin, Ji-Eun Bae, Hyun-Wook Choi, Moo Yeol Baik

**Affiliations:** 1 Department of Food Science and Biotechnology, Institute of Life Science and Resources, Graduate School of Biotechnology, Kyung Hee University, Yongin 17104, Republic of Korea; qew0226@gmail.com (S.-E.Y.); lifesci2015@khu.ac.kr (S.-J.Y.); msyw617@khu.ac.kr (M.-S.K.); seonmin@kfri.re.kr (S.-M.O.); drumlover@naver.com (J.-S.S.); wise123@khu.ac.kr (J.-E.B.); 2 Food Processing Research Group, Korea Food Research Institute, Wanju-gun 55365, Republic of Korea; 3 Department of Food and Nutrition, Jeonju University, Jeonju 55069, Republic of Korea

**Keywords:** short-chain glucan aggregates (SCGAs), thermo-reversibility, resistant starch (RS), cooked rice, In vitro starch gel digestibility

## Abstract

To enhance the food applicability of SCGAs, this study investigated the thermo-reversible behavior of SCGAs after cooking and evaluated the in vitro digestibility of starch gels in a real food system, i.e., cooked rice. For the thermo-reversibility of SCGAs, the DSC double-helical melting enthalpy and relative crystallinity of SCGAs decreased after cooking but increased upon cooling. In addition, cooling SCGAs to 30 °C after cooking led to increased slowly digestible starch (SDS) and RS contents associated with a lower glycemic index. These results indicate that cooked SCGAs undergo rapid recrystallization during the cooling process and maintain their resistance to digestion. For application in a real food system, RS content increased with the increasing amount of SCGAs. Furthermore, when the cooked rice was frozen and thawed, the RS content further increased due to the retrogradation of both cooked rice and SCGAs during storage. The appearance and textural characteristics of the cooked rice were not affected by adding SCGAs up to 20%. Nevertheless, the addition of SCGAs to the rice positively increased SDS and RS contents as well as reducing the estimated glycemic index (eGI), indicating low digestibility of starch gels. Consequently, SCGAs exhibited unique thermo-reversibility and low digestibility, which could be applied to real food systems. Overall, this study highlights the potential of SCGAs as a functional material for a wide range of thermally processed starch gel foods.

## 1. Introduction

Rice (*Oryza sativa* L.) is known to be the major source of carbohydrates for more than half of the world’s population, and while it is widely used in rice cakes, noodles, bread, and commercial foods, it is primarily consumed in the form of steamed rice, which is cooked with water to make starch gel and consumed [1]. One of the major reasons for rice consumption is its high starch content (72–82%) compared to other grains such as corn, wheat, and barley [2]. Refined rice contains about 90% starch, and cooked rice is generally categorized as a high glycemic index (GI) food [3]. There are many reports that regular consumption of a high glycemic index diet is related to an increased risk of chronic diseases such as diabetes and obesity [4]. Therefore, there have been many recent attempts to develop rice with a lower GI [5,6].

Traditionally, the GI is measured in-vivo, but enzymatic methods have also been developed to measure hydrolyzed glucose in vitro, which correlate with in vivo results [7,8]. Briefly, using in vitro enzymatic methods, starches in foods can be categorized into three groups: rapidly digestible starch (RDS), slowly digestible starch (SDS), and resistant starch (RS). RDS is quickly digested in the small intestine, causing an immediate rise in blood sugar. Conversely, SDS takes longer to fully digest and is associated with stable glucose metabolism [9]. RS is resistant to digestion in the small intestine but undergoes fermentation by microorganisms in the colon. Its hypoglycemic and probiotic effects may contribute to the prevention and functional regulation of adult diseases [10,11].

RS is considered beneficial in the human diet because it has effects similar to specific dietary fibers while also providing excellent texture and sensory characteristics. RS has been classified into five subtypes, denoted from RS1 to RS5, based on its nature: RS1 is physically inaccessible starch, RS2 is nongelatinized native starch granules, RS3 is retrograded or crystalline nongranular starch in starch gels, RS4 is chemically modified starch, and RS5 is amylose-lipid complexed starch [12]. In particular, RS3 is of great interest due to its higher thermal stability compared to other types of resistant starch, making it stable to heat treatment and allowing it to remain nondigestible in most food processes. RS3 can be prepared through physical treatments such as autoclaving and heat-hydration, as well as through acid and enzymatic hydrolysis [13].

A short-chain glucan aggregate (SCGA) is a type of RS3, produced from starch gels through enzymatic debranching and self-assembly of amylopectin [14]. A SCGA is a starch derivative, which can be prepared from the debranching of amylopectin and self-assembly of debranched short-chain glucans (SCGs). This SCGA has a relatively small size compared to starch granules, and debranched SCGs are recrystallized during aggregation. Therefore, the formation of SCGAs and the recrystallization of SCGAs occur simultaneously, resulting in a more dense structure, which provides reduced digestibility compared to gelatinized starch gels [15,16,17,18]. Starch nanoparticles (SNPs), including SCGAs, can optimize desired properties by controlling factors such as temperature, moisture, pH, and more. They offer the benefits of environmentally friendly manufacturing methods, low cost, and complete biodegradability [19]. Cai and Shi [20] prepared materials with type A and type B isomorphs, characterizing their properties and digestibility by manipulating the solids concentration and crystallization temperature of debranched starch. Recently, the unique thermo-reversible properties of SCGAs were revealed by Oh et al. [21]. SCGAs melted above 100 °C and recrystallized from 50 °C, distinct from natural starch, showing promise for applications in thermally processed foods.

To enhance the food applicability of SCGAs, specific studies on their post-cooking behavior and application in real food systems are required but limited information is available. Additionally, there are no studies that have added SCGAs to reduce the digestibility of starch gels in cooked rice. In this study, the physicochemical properties and in vitro digestibility of cooked SCGAs were investigated under different cooling temperatures. Additionally, freshly and freeze–thawed cooked rice were prepared by incorporating SCGAs at a specific ratio (0~20% *w*/*w*), and the cooking quality and estimated GI of starch gels in cooked rice were investigated.

## 2. Results and Discussion

### 2.1. Thermo-Reversibility of SCGAs

#### 2.1.1. Morphology

**Figure 1:**
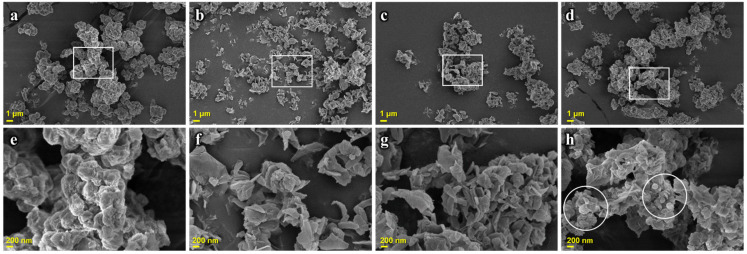
SEM images of native (**a**,**e**) and cooked SCGAs: (**b**,**f**), CS-100; (**c**,**g**), CS-60; (**d**,**h**), CS-30.

The morphologies of SCGAs before and after cooking were observed using scanning electron microscopy (SEM) (Figure 1). Native SCGAs showed micro-sized spherical aggregates before heating. The shape and size of SCGAs are influenced by factors such as starch and enzyme concentrations, self-assembly time, and temperature. SNPs produced from potatoes were primarily composed of spherical particles in the range of 15–30 nm, and when produced at a high starch concentration (7.5%), relatively large particles (50–120 nm) were observed [22]. Additionally, SCGAs self-assembled at high temperatures (50 °C) formed spherical nano-particles with a smaller size range compared to those crystallized at low temperatures (4 °C) [23].

On the other hand, morphological changes were observed in cooked and dried SCGAs at different temperatures. CS-100 and CS-60 showed individual particles with a plate-like structure. Additionally, smaller, irregularly shaped aggregates were formed instead of spherical aggregates. It has been reported that SCGAs formed spherical aggregates regardless of the number of heating cycles [21]. This discrepancy might come from the different drying method. In this study, the ethanol dehydration method was used to preserve the structure of the SCGAs at a specific temperature. Describing the structures immediately after heating, CS-100 suggests partial melting of SCGAs due to the heating process. The incomplete formation of particles in CS-60 indicates that the recrystallization time was insufficient. CS-30 was similar to native SCGAs, suggesting that the melted SCGAs during the heating process can recrystallize under specific conditions, leading to the retrogradation of SCGAs. Cai and Shi [24] reported that the retrogradation of amylose chains obtained from debranched waxy corn starch can lead to the well-developed formation of spherical structures. SCGAs were cooled to 25 °C after heating and then dried in an oven at 40 °C. In the presence of sufficient moisture, SCGAs can rearrange and retrograde during oven drying [25]. Gong et al. [26] reported that a short retrogradation period forms very small particles (diameter 10–30 nm), while an extended retrogradation period of over 120 min accelerates the growth of the mentioned nuclei, creating regular and larger particles (30–50 nm).

#### 2.1.2. X-Ray Diffraction (XRD) Patterns

**Figure 2:**
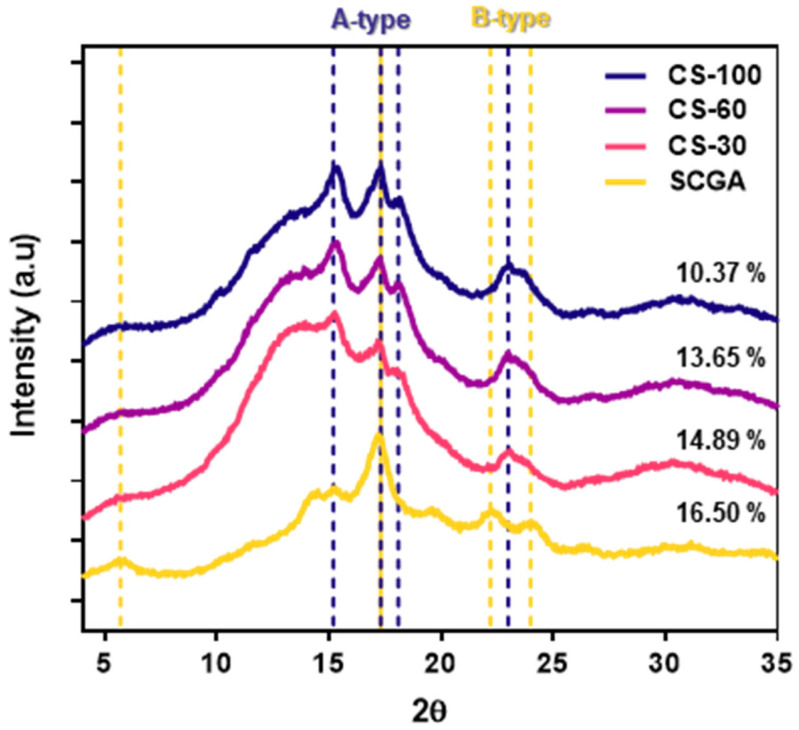
X-ray diffraction patterns and relative crystallinity of the SCGA before and after cooking.

The XRD patterns and relative crystallinity of the samples are shown in Figure 2. Native SCGAs exhibited a typical B-type crystalline structure with major peaks at 5.7°, 17.2°, 22.5°, and 24.3° (2θ). Miao et al. [27] reported that raw maize starch has an A-type crystalline structure, but when debranched starches were recrystallized at 4 °C, it transformed into a B-type crystalline structure. Furthermore, after debranching and aggregation, typical B-type structures were observed in waxy rice, waxy maize, and waxy potato starches [26].

The cooked SCGA showed an A-type crystalline structure with major peaks at 15.3°, 17.2°, 18.1°, and 23° (2θ). Generally, high-temperature incubation induced an A-type crystalline structure, while a B-type crystalline structure formed at low-temperature incubation [23,28]. The native SCGA was crystallized at 4 °C for 24 h, while the cooked SCGA underwent recrystallization at room temperature for a short duration. Additionally, the immediate removal of water using anhydrous ethanol may have influenced the formation of the A-type crystalline structure. Qiu et al. [29] reported that the combination of high temperature and ethanol addition to debranched amylose stimulated the formation of an A-type crystalline structure.

The relative crystallinity of the native SCGA was 16.50%, and significantly decreased to 10.37% immediately after cooking at 100 °C (CS-100). This could be attributed to the disruption and/or dissociation of the double-helical structure of the SCGA due to the heating process. However, the relative crystallinity of the cooked SCGA gradually increased with decreasing cooling temperatures, reaching 13.65% at 60 °C (CS-60) and 14.89% at 30 °C (CS-30). The relative crystallinity of the boiled or autoclaved SCGA was observed to be slightly higher or lower than the native SCGA, depending on the plant source [21]. Therefore, these results suggest that the melted double helices rearranged during the cooling process, forming a denser crystalline structure, resulting in relatively higher crystallinity.

#### 2.1.3. Thermal Properties

**Figure 3:**
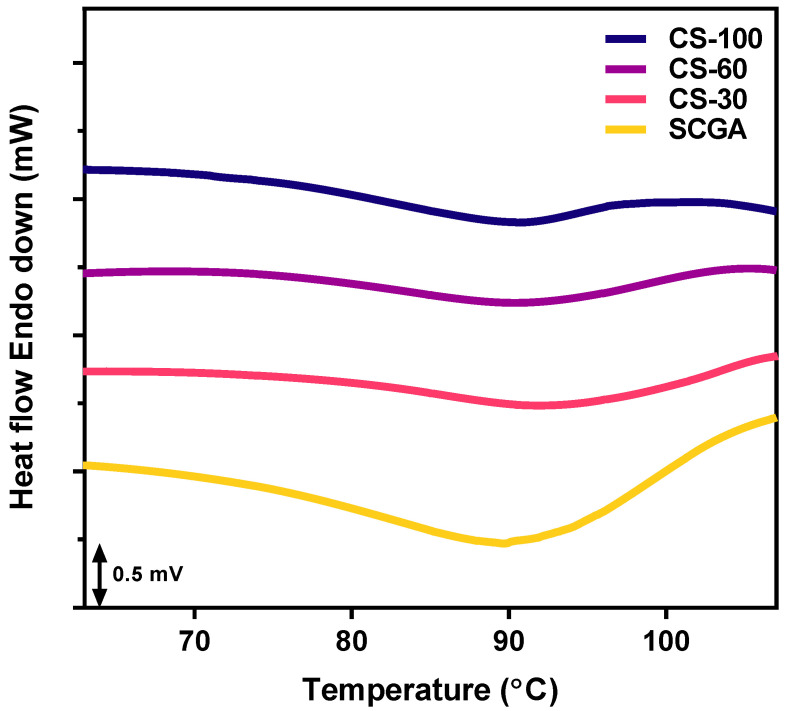
DSC thermogram of SCGAs before and after cooking.

The DSC thermograms and the thermal parameters of the SCGA are shown in Figure 3. The native SCGA showed a broad endothermic peak, while the thermograms of cooked SCGA revealed relatively narrow endothermic peaks. The cooked SCGA exhibited a higher onset temperature (T_o_) and a reduced double-helical melting transition range (T_c_ − T_o_) compared to those of the native SCGA. However, peak (T_p_) and conclusion temperatures (T_c_) did not change significantly (Table 1). The cooked SCGA revealed an A-type crystal, and the native SCGA showed a B-type crystal (Figure 2). Generally, T_o_ for A-type crystals is known to be higher than that for B-type crystals due to their relatively compact crystal structure [30]. The differences in T_c_ − T_o_ can be caused by the presence of crystallites, composed of small crystals with slightly different strengths [31]. Generally, the double-helical melting transition temperature range (T_c_ − T_o_) represents the homogeneity of the crystal structure. A narrow endothermic transition indicates a relatively homogeneous crystal structure and a broad endothermic transition represents a relatively heterogeneous crystal structure. Therefore, a decrease in T_c_ − T_o_ indicates the formation of more uniform crystals. In this study, ethanol precipitation was used to recover the recrystallized product at a specific temperature. During melting and recrystallization, most of the water-soluble materials were lost in the supernatant [32]. The removal of soluble short chains led to a more uniform chain length distribution, resulting in the formation of smaller and denser crystals and, consequently, a reduced transition range.

The change in double-helical melting enthalpy (ΔH) of the cooked SCGA significantly decreased compared to that of the native SCGA. This result is attributed to the reduced formation of the double helix due to the short recrystallization time and removal of soluble short-chain glucans. Previous studies have reported an increase in the perfection of crystals with an increase in the recrystallization time of debranched waxy maize starch [27].

Interestingly, the double-helical melting enthalpy (ΔH), immediately decreased after cooking, and gradually increased with decreasing temperatures (from 6.9 J/g at 100 °C to 7.7 J/g at 60 °C and 9.1 J/g at 30 °C, respectively). The double-helical melting temperature range of the native SCGA was 69.6 °C to 105.3 °C, suggesting that some double helices of the SCGA might melt during the cooking process. But the melted short-chain glucans interacted and rearranged during the cooling process and then formed thermo-stable double helices rapidly. The native SCGA showed the highest ΔH and the relative crystallinity and CS-100 revealed the lowest ΔH and the relative crystallinity. Consequently, the ΔH change was similar to the relative crystallinity change, which confirmed the thermo-reversible properties of the SCGA.

#### 2.1.4. In Vitro Digestibility

The in vitro digestibility of starch gels in the native SCGA and cooked SCGA is shown in Table 2. Considering the human health benefits of SDS and RS, many studies have aimed to increase the SDS and RS contents of natural starches [33,34]. Among them, the debranching and crystallization treatment is a cost-effective and safe method. The linear short-chain glucan (SCG) produced through enzymatic hydrolysis generates new double helices during retrogradation, expanding the crystalline regions and increasing the SDS and RS contents [35].

The RS content of the native SCGA (42.5%) was significantly higher than that of native waxy corn starch (7.3%). It is interesting to note that the RS content in CS-100 and CS-60 decreased compared to the untreated samples, while that in CS-30 increased (Table 2). The main reason for the highest RS content in CS-30 would be the recrystallization time. In the case of CS-100 and CS-60, they do not have enough time to recrystallize, while CS-30 has relatively enough time to recrystallize. After cooking, SCGAs were dissociated and they need time to recrystallize at a specific temperature. According to Oh et al. [14], the recrystallization kinetics of SCGAs are significantly dependent on temperature and time. In the case of starch gels, the RDS content decreased with cooling, while the sum of the SDS and RS content increased. It has been reported that heating and cooling treatments contribute to reducing the in vitro digestibility of starch gels by forming stable double helices and crystals [36]. Zeng et al. [37] repeatedly gelatinized and crystallized debranched waxy rice starch (DWRS), resulting in an increase in SDS content from 36.2% to 57.8%. After cooking, a decrease in RDS and RS and an increase in SDS content were observed. These changes could be associated with the proportion of amorphous and ordered packing regions. RDS is primarily located in the outer regions and has an amorphous structure [27]. SDS is located mostly in amorphous regions, and its small portion is present in the imperfect crystallites in the intermediate region between RDS and RS. On the other hand, RS has an ordered double-helix structure in the inner regions. Therefore, it seems that the helical structure of the crystalline region was partially loosened through heating, leading to imperfect packing and increased SDS content. Furthermore, relatively lower digestibility has been reported in the A-type crystalline structure compared to the B-type crystals in recrystallized short linear glucans because A-type aggregates form a dense structure, limiting accessibility to enzymes [28].

Consequently, the cooked SCGA exhibited lower relative crystallinity and double-helical melting enthalpy (ΔH) compared to the native SCGA. However, increased SDS and RS content were observed in the cooked SCGA, which was associated with a lower glycemic index (GI). In the case of the in vitro digestibility of starch gels, it is commonly believed that high ΔH and relative crystallinity correlate with high RS content, but this is not always true. When compared to normal or waxy corn starch gels, high-amylose corn starch gel had lower crystallinity but exhibited higher resistance to enzymatic digestion [38]. This study confirmed that SCGAs exhibited thermo-reversibility, which is a distinguished property compared to natural starch. This thermo-reversibility of SCGAs enhanced resistance to the in vitro digestibility of starch gels, suggesting a possible candidate for lowering GI in starch gel foods.

### 2.2. Application of SCGAs in Cooked Rice

#### 2.2.1. Appearance of Cooked Rice

**Figure 4:**
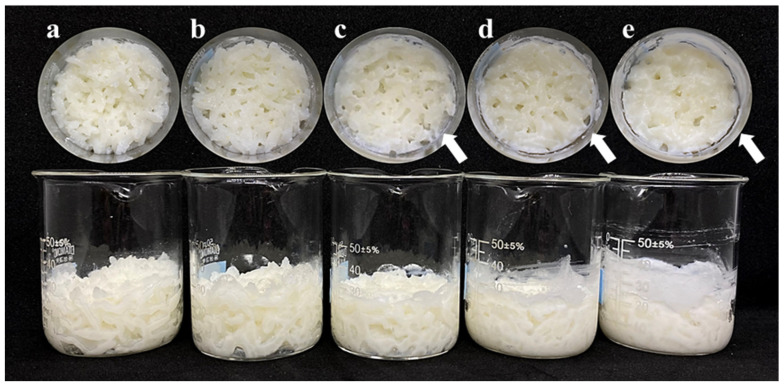
Appearance of cooked rice with (**a**) 10%, (**b**) 20%, (**c**) 30%, (**d**) 40%, and (**e**) 50% SCGA.

To evaluate the practical application of SCGAs in real food systems, cooked rice containing SCGAs was prepared. Prior to the main experiment, a preliminary experiment was conducted to determine the maximum level of SCGA addition that would not negatively affect the quality. Figure 4 shows the appearance of cooked rice with the SCGA replaced from 10% to 50% of the rice grain. When the SCGA was added at 10% and 20%, there was no noticeable impact on appearance and sensory characteristics. However, when it was added at 30% or more, the formation of a white residue on the surface was observed, possibly due to the presence of excess SCGA, which melted during the cooking process, and some of it aggregated or formed gels on the surface. Moreover, those consuming cooked rice with more than 30% added SCGA perceived dryness and a powdery mouthfeel, which could negatively impact consumer acceptability. Therefore, in this study, the maximum suitable addition of SCGA was chosen as 20%.

**Figure 5:**
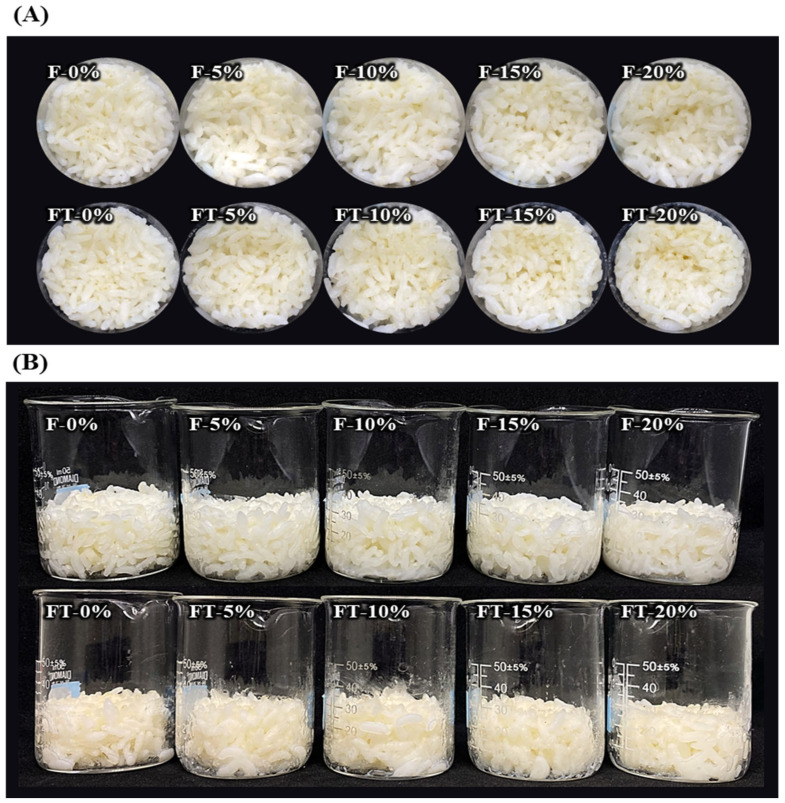
Top (**A**) and side (**B**) appearance of cooked rice with SCGA ranging from 0% to 20%. F: fresh cooked rice; FT: frozen and thawed cooked rice.

The appearance of cooked rice with SCGA from 0% to 20% is shown in Figure 5. No significant negative changes in appearance and sensory characteristics were observed in all samples. Although there was a tendency for increased hardness or slight volume reduction in frozen and thawed samples, the extent was not significant.

#### 2.2.2. Morphology of Cooked Rice

**Figure 6:**
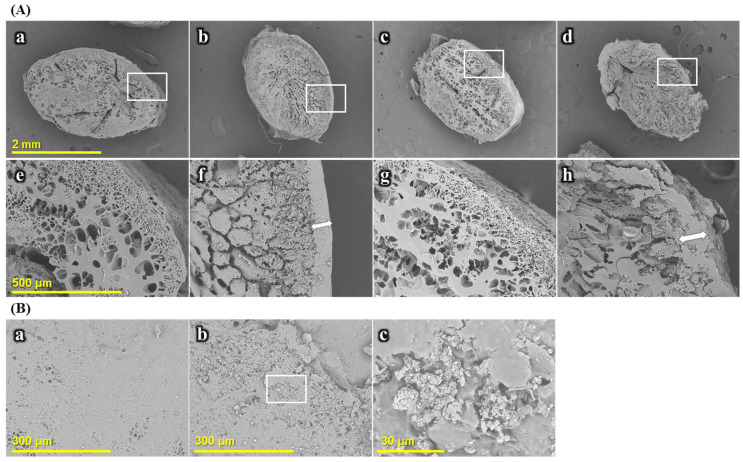
Cross-section images of cooked rice grains (**A**): (**a**,**e**), F-0%; (**b**) and (**f**), F-20%; (**c**,**g**), FT-0%; (**d**,**h**), FT-20%; and surface images of cooked rice grains (**B**): (**a**), F-0%; (**b**) and (**c**), F-20% determined via SEM.

The morphological changes in the rice grains were observed based on cross-section and surface images (Figure 6). The microstructure of the rice grain was significantly affected by the addition of SCGA and the presence or absence of frozen storage. In the cross-section of fresh cooked rice without SCGA (Figure 6(Aa,Ae)), a honeycomb structure was clearly observed. When the rice grains are cooked, the internal structure of the rice amyloplast is partially arborized at 50 °C, and when the temperature exceeds 70 °C, the cell membrane of the amyloplast becomes loose and develops a honeycomb structure [39,40]. Therefore, gelatinization of starch granules during the cooking process significantly increases their susceptibility to enzymatic degradation. However, in the samples with 20% SCGA (Figure 6(Ab,Af)), a thick coated layer was formed on the surface of rice grains, and a honeycomb structure was not produced. Ha et al. [41] reported that the formation of the coated layer was attributed to the leaching of amylose during the gelatinization of rice starch. SCGA consists of short-chain glucans, which are relatively smaller than amylose. Small SCG has more mobility than amylose and can be easily rearranged. Moreover, the amount of SCG is much higher than the amylose content in rice, and this high amount of SCG plays an important role in the coating of rice grains. Amylose leaches out from the starch granule, but SCG is already prepared and added separately [14]. This result confirmed that the SCGA melting, rather than amylose leaching, had a greater influence on the formation of a coated layer on the surface of the rice grain when certain amounts of SCGA were added. The coated layer is expected to act as a physical barrier to digestive enzymes as well as cause incomplete gelatinization, thus affecting the digestibility of starch gels in cooked rice.

Frozen storage has been reported to affect the quality and digestibility of starch gels in cooked rice by influencing starch retrogradation and textural properties [42]. An increase in pore size was observed in the cross-section images of freeze–thaw samples (Figure 6(Ac,Ag)) compared to freshly cooked rice without SCGA. This is possibly due to the formation of ice crystals during frozen storage and their removal during the thawing process. In the FT-20% sample (Figure 6(Ad,Ah)), decreases in the thickness of the coated layer and the pore size were found. This is possibly due to the loss of moisture and weakening of the inner matrix during storage [43]. It has been reported that the retrogradation of gelatinized rice at low temperatures caused the breaking and disintegration of starch granules, resulting in the separation of water and starch molecules [44].

On the surface of the fresh cooked rice samples (Figure 6B), a smooth structure was observed without SCGA (Figure 6(Ba)). On the other hand, in the cooked rice with 20% SCGA (Figure 6(Bb,Bc)), partial aggregation of SCGA was observed along with a rough surface structure. Based on these results, it can be predicted that SCGA is partially melted during the cooking process to form a coated layer, and the remaining SCGA on the surface of the rice grains affects the texture and digestibility of the starch gels in cooked rice.

#### 2.2.3. In Vitro Digestibility of Starch Gels and Estimated Glycemic Index (eGI) of Cooked Rice

Table 3 shows the in vitro digestibility of starch gels in cooked rice with SCGA. The increase in SCGA amount in cooked rice resulted in a decrease in RDS content as well as an increase in SDS and RS contents, respectively. In particular, in the case of 20% addition of SCGA, the RS content increased more than ten-fold from 0.49% (F-0%) to 5.00% (F-20%). It is generally known that the addition of a type of soluble dietary fiber, such as oligosaccharides, to rice can reduce digestibility. It has been reported that guar gum governs gelatinization behavior by interacting with amylose and amylopectin on the surface of starch granules [45]. Oligosaccharides can form a network around starch granules, restricting the leaching of amylose chains during gelatinization and potentially limiting the access of digestive enzymes [46]. The observed formation of a coated layer due to the melting of SCGA is expected to play a significant role similar to the interaction between oligosaccharides and starch gels in cooked rice, acting as a barrier to the digestive enzymes.

**Figure 7:**
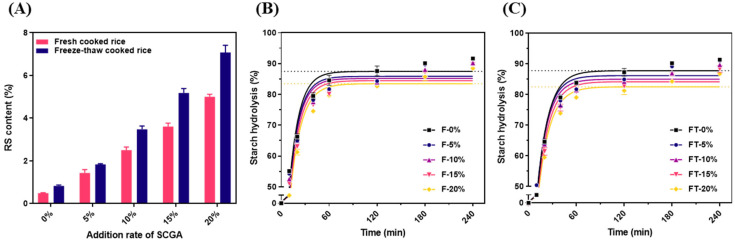
Effects of freeze–thaw treatment on the RS contents of cooked rice with SCGA (**A**), and starch gel hydrolysis rate of fresh (**B**) and freeze–thaw cooked rice with SCGA (**C**).

Interestingly, all freeze–thaw samples exhibited an increase in RS content compared to fresh cooked rice with or without SCGA. Without SCGA, the freeze–thaw sample (0.81%) showed a higher value than that of the fresh sample (0.49%). The increases in RS contents of cooked rice with SCGA were also more pronounced than those of fresh cooked rice (Figure 7A). This is possibly due to the synergistic effect of the retrogradation of rice starch gel and the recrystallization of SCGA during frozen storage. The RS content of cooked rice has been reported to be doubled after refrigerated storage, regardless of the grain type [47]. Refrigerated rice showed higher SDS content than that of frozen rice, and the rice cooker method formed a more perfect crystalline structure during storage, resulting in higher RS content. An in vivo study suggested that consuming reheated rice after refrigerated storage reduces the long-term risk of type 2 diabetes compared to freshly cooked or partially cooked rice [5]. RS is considered a specific dietary fiber, which is beneficial to the human diet, while it provides excellent texture and sensory characteristics in food systems. RS is known to be digestible in the small intestine but fermented by microorganisms in the colon. Therefore, the hypoglycemic and probiotic properties of RS contribute to the prevention and functional regulation of adult diseases [10,11].

Figure 7B,C show the starch gel hydrolysis rate of fresh and freeze–thaw cooked rice with SCGA. All four parameters associated with starch gel digestibility, such as C_∞_, *k*, HI, and eGI, significantly decreased with increasing SCGA amount. It has been reported that the addition of polysaccharides to rice can lead to the formation of a network with starch granules that is difficult to hydrolyze by digestive enzymes, resulting in a reduced GI [48]. As mentioned in the previous section, the addition of SCGA forms a coated layer on the surface of rice grains. This coated layer acts as a physical barrier, preventing the leaching of amylose during gelatinization and the enzymatic hydrolysis of starch gels, resulting in a relatively low digestibility.

Additionally, when the same amount of SCGA was added, freeze–thaw cooked rice revealed lower C_∞_ and eGI values than those of fresh cooked rice. It has been reported that consuming freshly cooked rice after cooling (whether reheated or not) resulted in a lower postprandial glycemic response [5,49]. Moreover, the starch hydrolysis rate of microwave-reheated cooked rice after 24 h of storage was reported to be slightly lower than that of fresh cooked rice [50]. Freshly cooked and cooled SCGAs do not have enough time to retrograde and provide relatively high digestibility. However, freeze–thaw treatment provides relatively more time to retrograde or recrystallize and gives relatively low digestibility. During freeze–thaw treatment, starch gels in rice and SCGA molecules were rearranged to a more ordered and recrystallized structure, resulting in reduced digestibility.

Consequently, the slow-digestion characteristics of cooked rice with SCGA were confirmed in this work. The slow digestion characteristic of SCGA was maintained in the real food even after cooking, freezing, and reheating. These results suggest the potential of SCGA as a resistant starch to a variety of products with or without thermal processing. In particular, it is expected that SCGA could be more effective in reducing the digestibility of starch gels when applied to the retort-processed foods that undergo cooking and reheating.

## 3. Conclusions

In this study, the unique thermo-reversible properties of SCGA and its suitability for food applications were discovered. To investigate the thermo-reversibility of SCGA, the physicochemical properties and digestibility of cooked SCGA collected at different temperatures during the cooling process were evaluated. DSC and X-ray diffraction analysis of cooked SCGA revealed that the double-helical melting enthalpy and relative crystallinity decreased immediately after cooking and increased upon cooling. These results indicate that cooked SCGA undergoes rapid recrystallization during the cooling process. It was observed that the cooking and cooling of SCGA increased the SDS and RS contents compared to native SCGA, suggesting the possible application of SCGA in thermally processed food systems as a resistant starch. When SCGA was added to rice and cooked together, the appearance and textural characteristics of the cooked rice were not affected by adding SCGA up to 20%. The RS content of cooked rice increased with the increasing amount of SCGA. Furthermore, when the cooked rice was frozen and thawed, the RS content further increased due to the retrogradation of both rice starch gels and SCGA during storage. Starch gels in rice and SCGA molecules were rearranged to a more ordered and recrystallized structure during freeze–thaw treatment, resulting in reduced digestibility. The addition of SCGA to the rice positively reduced the starch gel hydrolysis rate (HI) and estimated glycemic index (eGI). These results confirmed that SCGA maintained its resistance to digestion even in cooked foods. Consequently, this work confirmed the thermo-reversible properties of SCGA, and its potential use for food applications has been established. It is expected that SCGA can be successfully utilized as a functional material in various types of thermally processed retort or canned starchy foods as a resistant starch to help diabetic people.

## 4. Materials and Methods

### 4.1. Materials

Waxy corn starch was obtained from Samyang Genex Co. (Seoul, Republic of Korea). Pullulanase (Promozyme D2; 1350 NPUN/g, 1.15 g/mL) was purchased from Sigma-Aldrich Co. (St. Louis, MO, USA). The Digestible and Resistant Starch Assay Kit (K-DSTRS) was obtained from Megazyme International Ireland Ltd. (Wicklow, Ireland). Polished rice grain (Shin-dongjin, japonica white rice, Jeonju, Republic of Korea) was purchased from a local store.

### 4.2. Preparation of Short-Chain Glucan Aggregates (SCGAs)

SCGAs were prepared according to the method of Oh et al. [14], with some modifications. Waxy corn starch (30 g) was dissolved in 20 mM sodium acetate buffer (pH 5.0, 300 mL), and the dispersion was gelatinized in boiling water for 30 min with stirring. Samples were cooled down to 60 °C, and then pullulanase (60 μL/g starch) was added and incubated at 60 °C for 12 h. The solution was stored at 4 °C for 12 h for self-assembly. After self-assembly, the SCGA was collected, washed 3 times with distilled water, dried at 40 °C, ground using a mortar, and sieved through a 100-mesh sieve. The yield of SCGA was calculated using the following equation:

$$Yield\;\left(\%\right)\;=\;\frac{Weight\;of\;SCGA}{Weight\;of\;initial\;waxy\;corn\;starch}\;\times \;100$$

### 4.3. Thermo-Reversibility of SCGA

#### 4.3.1. Preparation of Cooked SCGA

To investigate the post-cooking behavior of SCGA, the SCGA was reheated and then cooled. Cooked SCGA was cooled at specific temperatures (100 °C, 60 °C, and 30 °C) and collected to observe changes in physicochemical properties and in vitro digestibility during the cooling process.

Specifically, SCGA was dispersed in distilled water (5%, *w*/*v*) and cooked in boiling water with stirring for 30 min. After cooking (100 °C) or upon cooling to 60 °C and 30 °C, anhydrous ethanol was added to SCGA for dehydration. Ethanol dehydration is an effective method to preserve starch’s molecular structure at a specific point in time by preventing further retrogradation [51]. The samples were then centrifuged at 3200 rpm for 10 min, and the precipitate was dried, ground, and sieved using a 100-mesh sieve. The cooked SCGA was named CS-100, CS-60, and CS-30, corresponding to the cooling temperatures.

#### 4.3.2. Morphology

To investigate the morphologies of SCGA at different temperatures during the cooling process, the native SCGA and cooked SCGA were observed with a high-resolution scanning electron microscopy device (HR-SEM, Merlin, Carl Zeiss, Oberkochen, Germany). All samples were put on a double-sided carbon tape and coated using a gold-palladium (60:40). The images were observed at an accelerating voltage of 5 kV.

#### 4.3.3. X-Ray Diffraction Patterns

The X-ray diffraction pattern of SCGA was obtained using an X-ray diffractometer (D8 Advance, Bruker AXS GmbH, Karlsruhe, Germany). The X-ray diffraction of the sample was recorded from 4–35° (2θ) at a rate of 6°/min, 40 kV voltage, and 40 mA. The relative crystallinity was calculated as the ratio of the crystalline area to the total diffraction area from the XRD pattern.

#### 4.3.4. Thermal Properties

Thermal properties were analyzed using a differential scanning calorimeter (DSC 4000, Perkin Elmer Inc., Waltham, MA, USA). An SCGA slurry (75% moisture content, *w*/*w*) was placed into an aluminum pan and sealed, with an empty pan used as a reference. The samples were scanned from 25 °C to 150 °C at a rate of 10 °C/min. The onset (T_o_), peak (T_p_), and conclusion (T_c_) temperatures and enthalpy (ΔH) of double-helical melting transition were obtained from the DSC thermogram using Pyris software (version 13.3).

#### 4.3.5. In Vitro Digestibility

In vitro digestibility was determined using the Megazyme Digestible and Resistant Starch Assay Kit according to the method developed by McCleary et al. [52] with slight modifications. Briefly, the sample (0.5 g) was dissolved in sodium maleate buffer (17.5 mL, 50 mM, pH 6.0) and incubated in a water bath at 37 °C for 5 min. After equilibration, a mixture of pancreatic α-amylase and amyloglucosidase (2.5 mL) was added, followed by incubation at 37 °C with stirring. An aliquot (1.0 mL) was taken at 20 min and 120 min and then acetic acid solution (20 mL) was added to inactivate the enzyme. At 120 min, 4.0 mL of an aliquot was taken, 4.0 mL of ethanol was added, and centrifuged. The precipitates were washed with ethanol and then suspended in NaOH to dissolve the nondigestible starch, facilitating the measurement of total starch (TS). The amount of released D-glucose was measured using a glucose oxidase and peroxidase reagent (GOPOD). The RDS, SDS, and RS contents were expressed as % (d.b.) of the TS in the sample and calculated using the following equations:

$$Rapidly\;digestbility\;starch\;(RDS,\%)\;={(G}_{20}\;\times \;F\;\times \;0.9)/W\;\times \;100$$

$$Slowly\;digestbility\;starch\;(SDS,\%)\;=\;{((G}_{120}{\;-\;G}_{20})\;\times \;F\;\times \;0.9)/W\;\times \;100$$

$$Slowly\;digestbility\;starch\;(SDS,\%)\;=\;{((G}_{120}{\;-\;G}_{20})\;\times \;F\;\times \;0.9)/W\;\times \;100$$

$$Total\;starch\;(TS,\%)\;=\;{(G}_{TS}\;\times \;F\;\times \;0.9)/W\;\times \;100$$

$$Resistant\;starch\;\left(RS,\%\right)\;=\;TS\;-\;(RDS\;+\;SDS)$$

where G_TS_ = absorbance of total starch, G_20_ = absorbance after 20 min, G_120_ = absorbance after 120 min, F = 100/GOPOD absorbance, and W = sample weight (mg).

### 4.4. Application to Cooked Rice

#### 4.4.1. Preparation of Cooked Rice with or Without SCGA

To evaluate the practical application of SCGA in a real food system, cooked rice containing SCGA was prepared. The rice grains were substituted with 0, 5, 10, 15, and 20 g/100 g (*w*/*w*) of SCGA. The samples were mixed with water at a ratio of 1:1.2 (sample: water, *w*/*v*) and soaked at room temperature for 30 min. The soaked sample was then cooked in an electric rice cooker (CR-1010FB, Cuckoo, Seoul, Republic of Korea) using a standard cooking mode. Cooked rice was cooled to room temperature for 30 min to allow for moisture equilibration. The fresh cooked rice samples were named F-0%, F-5%, F-10%, F-15%, and F-20% according to the SCGA contents, respectively. To investigate the effect of freezing, cooked rice was put into a −20 °C refrigerator and stored for 5 days. The frozen rice was reheated in a microwave oven for 2 min before analysis and then cooled using the same method as the fresh sample. The freeze–thaw cooked rice samples were named as FT-0%, FT-5%, FT-10%, FT-15%, and FT-20% according to the SCGA contents, respectively.

#### 4.4.2. Morphology

A scanning electron microscope (SEM-TM3000, Hitachi, Japan) was used to observe the morphology of fresh and freeze–thaw cooked rice grains. Prior to microscopic analysis, the cooked rice was freeze-dried using a freeze dryer (PVTFD10R, Ilshin Biobase, Yangju, Republic of Korea) for 48 h. The surface and cross-section of the rice grain were observed using a whole grain and cut into the half of a whole grain using a razor blade, respectively. The samples were placed on double adhesive tape and coated with gold using a sputter coater, and the accelerating voltage used was 15 kV.

#### 4.4.3. In-Vitro Digestibility of Starch Gels and Estimated Glycemic Index (eGI)

To obtain powdered materials for starch fraction determination, some samples were freeze-dried, ground, and sieved using a 100-mesh sieve. To determine the estimated glycemic index (eGI) of the well-chewed sample, the cooked rice sample was homogenized using a household blender (SFM-C353NK, Shinil Industrial Co., Ltd., Cheonan, Republic of Korea) for 2 min. The slurry state samples are typically used to determine the digestibility of starch gels in real food [7].

The in vitro digestibility of starch gels in cooked rice was determined according to the method of Section 4.3.5. During the enzymatic reaction, aliquots were withdrawn at 10, 20, 40, 60, 120, 180, and 240 min to calculate the starch hydrolysis rate over time, respectively. The kinetics of starch hydrolysis and the estimated glycemic index (eGI) were determined using the following equations [8]:

$$C\;=\;C_{\infty }\left(1\;-\;e^{-kt}\right)$$

$$AUC\;=\;C_{\infty }t\;-\;\frac{C_{\infty }}{k}\left(1\;-\;e^{-kt}\right)$$

eGI = 39.71 + 0.549HI

where C represents the percentage of starch hydrolyzed at time t (min), C_∞_ is the equilibrium concentration of hydrolyzed starch, and *k* is the kinetic constant. The area under the hydrolysis curve (AUC) can be integrated using the above equation. Based on the AUC, the hydrolysis index (HI) was calculated as the percentage of the sample’s AUC corresponding to the AUC of the reference food (white bread).

### 4.5. Statistical Analysis

All experiments were performed at least three times. The results are represented as the mean ± standard deviation. Starch hydrolysis curves and their parameters were analyzed using GraphPad Prism software version 8.4.3 (GraphPad Prism Inc., San Diego, CA, USA). Statistical analysis was carried out using analysis of variance (ANOVA), followed by Tukey’s test using SAS software version 9.4 (SAS Institute Inc., Cary, NC, USA).

## Figures and Tables

**Table 1:** DSC thermal properties of the SCGA before and after cooking.

Sample	T_o_ (°C)	T_p_ (°C)	T_c_ (°C)	T_c_ − T_o_ (°C)	∆H (J/g)
SCGA	69.6 ± 2.2 ^b^*	91.2 ± 1.3 ^b^	105.3 ± 0.9 ^a^	35.7 ± 2.9 ^a^	27.8 ± 1.3 ^a^
CS-100	76.0 ± 0.7 ^a^	91.6 ± 1.1 ^ab^	100.2 ± 1.8 ^b^	24.2 ± 2.5 ^b^	6.9 ± 0.6 ^c^
CS-60	74.8 ± 0.4 ^a^	91.4 ± 1.0 ^b^	103.1 ± 0.6 ^ab^	28.3 ± 1.0 ^b^	7.7 ± 0.5 ^c^
CS-30	75.8 ± 0.5 ^a^	94.7 ± 1.3 ^a^	105.4 ± 1.3 ^a^	29.6 ± 1.5 ^b^	9.1 ± 0.6 ^b^

* Values with the different letters in the same column are significantly different (*p* < 0.05).

**Table 2:** In vitro digestibility of SCGAs before and after cooking.

Sample	RDS (%)	SDS (%)	RS (%)	SDS + RS (%)
SCGA	30.9 ± 1.2 ^a^*	26.6 ± 1.5 ^c^	42.5 ± 2.2 ^a^	73.3 ± 1.2 ^c^
CS-100	24.9 ± 0.7 ^b^	43.7 ± 2.9 ^a^	31.4 ± 2.5 ^b^	75.1 ± 0.7 ^b^
CS-60	22.6 ± 1.9 ^bc^	41.2 ± 0.7 ^a^	36.1 ± 1.4 ^b^	77.3 ± 1.9 ^ab^
CS-30	19.9 ± 2.7 ^c^	32.6 ± 2.1 ^b^	47.5 ± 2.6 ^a^	80.1 ± 2.7 ^a^

* Values with the different letters in the same column are significantly different (*p* < 0.05).

**Table 3:** Starch fractions and hydrolysis kinetics of fresh and freeze–thaw cooked rice with different addition rates of SCGA.

Addition Rate (%)	Starch Fractions			Hydrolysis Kinetics			
	RDS (%)	SDS (%)	RS (%)	C_∞_ (%) *	*k* (min^−1^) *	HI *	eGI *
Fresh cooked rice							
0	97.63 ± 0.20 ^a^**	1.87 ± 0.21 ^d^	0.49 ± 0.02 ^f^	87.5 ± 0.7 ^a^	0.082 ± 0.003 ^a^	88.8 ± 0.6 ^a^	88.4 ± 0.3 ^a^
5	96.12 ± 0.23 ^bc^	2.44 ± 0.27 ^cd^	1.44 ± 0.16 ^e^	85.9 ± 0.3 ^bc^	0.082 ± 0.001 ^a^	87.0 ± 0.1 ^bc^	87.4 ± 0.1 ^bc^
10	94.33 ± 0.24 ^de^	3.16 ± 0.35 ^bc^	2.51 ± 0.14 ^d^	85.2 ± 0.3 ^bcd^	0.081 ± 0.001 ^ab^	86.2 ± 0.5 ^cd^	87.0 ± 0.2 ^cd^
15	92.48 ± 0.45 ^f^	3.95 ± 0.27 ^ab^	3.57 ± 0.20 ^c^	84.5 ± 0.2 ^d^	0.077 ± 0.001 ^bc^	85.2 ± 0.2 ^de^	86.4 ± 0.1 ^de^
20	90.59 ± 0.14 ^g^	4.41 ± 0.27 ^a^	5.00 ± 0.12 ^b^	83.3 ± 0.3 ^ef^	0.073 ± 0.001 ^cde^	84.0 ± 0.2 ^f^	85.7 ± 0.1 ^f^
Freeze–thaw cooked rice							
0	96.73 ± 0.19 ^ab^	2.44 ± 0.23 ^cd^	0.83 ± 0.05 ^f^	87.8 ± 0.6 ^a^	0.071 ± 0.002 ^de^	88.1 ± 0.6 ^ab^	88.0 ± 0.3 ^ab^
5	95.25 ± 0.30 ^cd^	2.91 ± 0.31 ^c^	1.84 ± 0.03 ^e^	86.2 ± 0.4 ^b^	0.075 ± 0.001 ^cd^	86.8 ± 0.5 ^c^	87.2 ± 0.3 ^c^
10	93.46 ± 0.27 ^ef^	3.06 ± 0.32 ^bc^	3.48 ± 0.16 ^c^	85.0 ± 0.1 ^cd^	0.074 ± 0.001 ^cd^	85.6 ± 0.1 ^d^	86.6 ± 0.0 ^d^
15	90.94 ± 0.47 ^g^	3.88 ± 0.48 ^ab^	5.18 ± 0.21 ^b^	84.2 ± 0.3 ^de^	0.070 ± 0.001 ^de^	84.4 ± 0.5 ^ef^	85.9 ± 0.3 ^ef^
20	88.46 ± 0.60 ^h^	4.46 ± 0.43 ^a^	7.10 ± 0.33 ^a^	82.5 ± 0.4 ^f^	0.069 ± 0.000 ^e^	82.5 ± 0.2 ^g^	84.9 ± 0.1 ^g^

* *C*_∞_, equilibrium starch gel hydrolysis concentration; *k*, rate coefficients; HI, hydrolysis index; eGI, estimated glycemic index. ** Values with different letters in the same column are significantly different (*p* < 0.05).

## Data Availability

The original contributions presented in this study are included in the article. Further inquiries can be directed to the corresponding authors.
